# A retrospective analysis based on multiple machine learning models to predict lymph node metastasis in early gastric cancer

**DOI:** 10.3389/fonc.2022.1023110

**Published:** 2022-12-01

**Authors:** Tao Yang, Javier Martinez-Useros, JingWen Liu, Isaias Alarcón, Chao Li, WeiYao Li, Yuanxun Xiao, Xiang Ji, YanDong Zhao, Lei Wang, Salvador Morales-Conde, Zuli Yang

**Affiliations:** ^1^ Department of Gastrointestinal Surgery, Guangdong Provincial Key Laboratory of Colorectal and Pelvic Floor Diseases, Guangdong Institute of Gastroenterology, The Sixth Affiliated Hospital of Sun Yat-sen University Guangzhou, Guangdong, China; ^2^ Unit of Innovation in Minimally Invasive Surgery, Department of General and Digestive Surgery, University Hospital “Virgen del Rocio”, Sevilla, Spain; ^3^ Translational Oncology Division, OncoHealth Institute, Health Research Institute - Fundacion Jimenez Diaz, Madrid, Spain; ^4^ Area of Physiology, Department of Basic Health Sciences, Faculty of Health Sciences, Rey Juan Carlos University, Madrid, Spain; ^5^ Shenzhen Institutes of Advanced Technology, Chinese Academy of Sciences, Shenzhen, Guangdong, China; ^6^ Faculty of Medicine, Autonomous University of Madrid, Madrid, Spain; ^7^ Department of Pathology, The Sixth Affiliated Hospital, Sun Yat-Sen University, Guangzhou, China

**Keywords:** early gastric cancer, endoscopic resection, gastrectomy, lymph node metastasis, artificial intelligence, machine learning

## Abstract

**Background:**

Endoscopic submucosal dissection has become the primary option of treatment for early gastric cancer. However, lymph node metastasis may lead to poor prognosis. We analyzed factors related to lymph node metastasis in EGC patients, and we developed a construction prediction model with machine learning using data from a retrospective series.

**Methods:**

Two independent cohorts’ series were evaluated including 305 patients with EGC from China as cohort I and 35 patients from Spain as cohort II. Five classifiers obtained from machine learning were selected to establish a robust prediction model for lymph node metastasis in EGC.

**Results:**

The clinical variables such as invasion depth, histologic type, ulceration, tumor location, tumor size, Lauren classification, and age were selected to establish the five prediction models: linear support vector classifier (Linear SVC), logistic regression model, extreme gradient boosting model (XGBoost), light gradient boosting machine model (LightGBM), and Gaussian process classification model. Interestingly, all prediction models of cohort I showed accuracy between 70 and 81%. Furthermore, the prediction models of the cohort II exhibited accuracy between 48 and 82%. The areas under curve (AUC) of the five models between cohort I and cohort II were between 0.736 and 0.830.

**Conclusions:**

Our results support that the machine learning method could be used to predict lymph node metastasis in early gastric cancer and perhaps provide another evaluation method to choose the suited treatment for patients.

## Introduction

Gastric cancer is one of the most common and deadly cancers in the world (1). According to GLOBOCAN 2021 data, gastric cancer is the third leading cause of cancer deaths worldwide, following only lung and liver cancers in overall mortality (2). Fortunately, because of the improvement in diagnosis and treatment, the survival rate for gastric cancer has been improved in recent years (1, 3, 4). Based on a report from the global surveillance of trends in cancer survival programs, age-standardized 5-year net survival for stomach cancer was below 30% in most countries, but high in Korea (69%) and Japan (60%), where it increased by up to 10% between 2000–2004 and 2010–2014; this is likely to be associated with endoscopic screening programs for early detection (5). Therefore, it is crucial to identify gastric cancer patients in the early stage.

Early gastric cancer (EGC) is defined as a stomach lesion confined to the mucosa and/or submucosa, regardless of its area or lymph node metastatic (LNM) status (6). Due to advances in endoscopic therapeutic techniques, the EGC has usually been diagnosed in the early detection and treated by endoscopic submucosal dissection (ESD) (7, 8). Many studies have shown that EGC has a 5-year survival rate of near 90% (9, 10). As the definition of EGC, the regional LNM is one of the most important prognostic factors in EGC. One report of trends in Incident, Management, and Survival in a Well-Defined French Population of Early Gastric Cancer demonstrated that the 5-year net survival was 50% in node-positive patients and 85% in node-negative patients (11). As a result, the lymph node positiveness decides the survival of EGC and whether the additional lymphadenectomy is required (12).

The previous studies confirmed that several risks such as tumor size, invasion depth, ulceration, histological types, and lymph vascular invasion were related with LNM in EGC (13–16). Even a few of research based on these factors constructed traditional scoring to evaluate the probability of LNM in EGC after the endoscopic resection (17, 18). According to the previous study, the percentage of actual lymph node positive after additional surgery of EGC is about 10% based on these scorings (19, 20). Certainty, the accuracy of these scorings is necessary more data of clinical practice.

Artificial intelligence (AI) is an advanced technology that has been used in many fields such as in industry, agriculture, navigation, driverless car, and healthcare (21–23). AI is a subfield of computer science that emphasizes the design of intelligent systems that can learn from the data and make decisions and predictions accordingly (24). Among many branches of AI, machine learning (ML) and deep learning (DL) are two major parts of all (25). ML is a mathematical AI algorithm automatically built from given data to predict precise outcomes in uncertain conditions without being explicitly programmed (26).

Currently, ML has been used to the wide area of medicine; the potential ability of ML can improve the efficiency and accuracy of clinical work, such as analyzing millions of clinical data to create prognostic, screening, and diagnostic models (27–29). ML has a satisfactory to excellent accuracy for predicting cancer, such as the oral cavity cancer; the accuracy prediction of cervical LNM was about 90% (30) and, in the early stage of colorectal cancer, ML model showed superior performance compared with conventional criteria in predicting LNM (31). In EGC, few studies have established predictive models with ML. For the reasons stated above, in the present multicenter study, we aim to study EGC with the additional surgery to evaluate the factors such as LNM better to construct a robust prediction model with ML to provide another evaluation method to choose the suited treatment for patients.

## Material and methods

### Study design

This was a multicenter, retrospective analysis. The cohort I was obtained from the Sixth Affiliated Hospital of Sun Yat-Sen University (Guangzhou, China), which was used to construct the prediction models, and the cohort II as the external validation date was from the University Hospital Virgen del Rocio (Seville, Spain), which was performed to verify the ability of models. The present study was approved by the Institutional Review Board of the Sixth Affiliated Hospital of Sun Yat-Sen University and the University Hospital Virgen del Rocio; the approval number is E2021197.

### Study population

**Figure 1:**
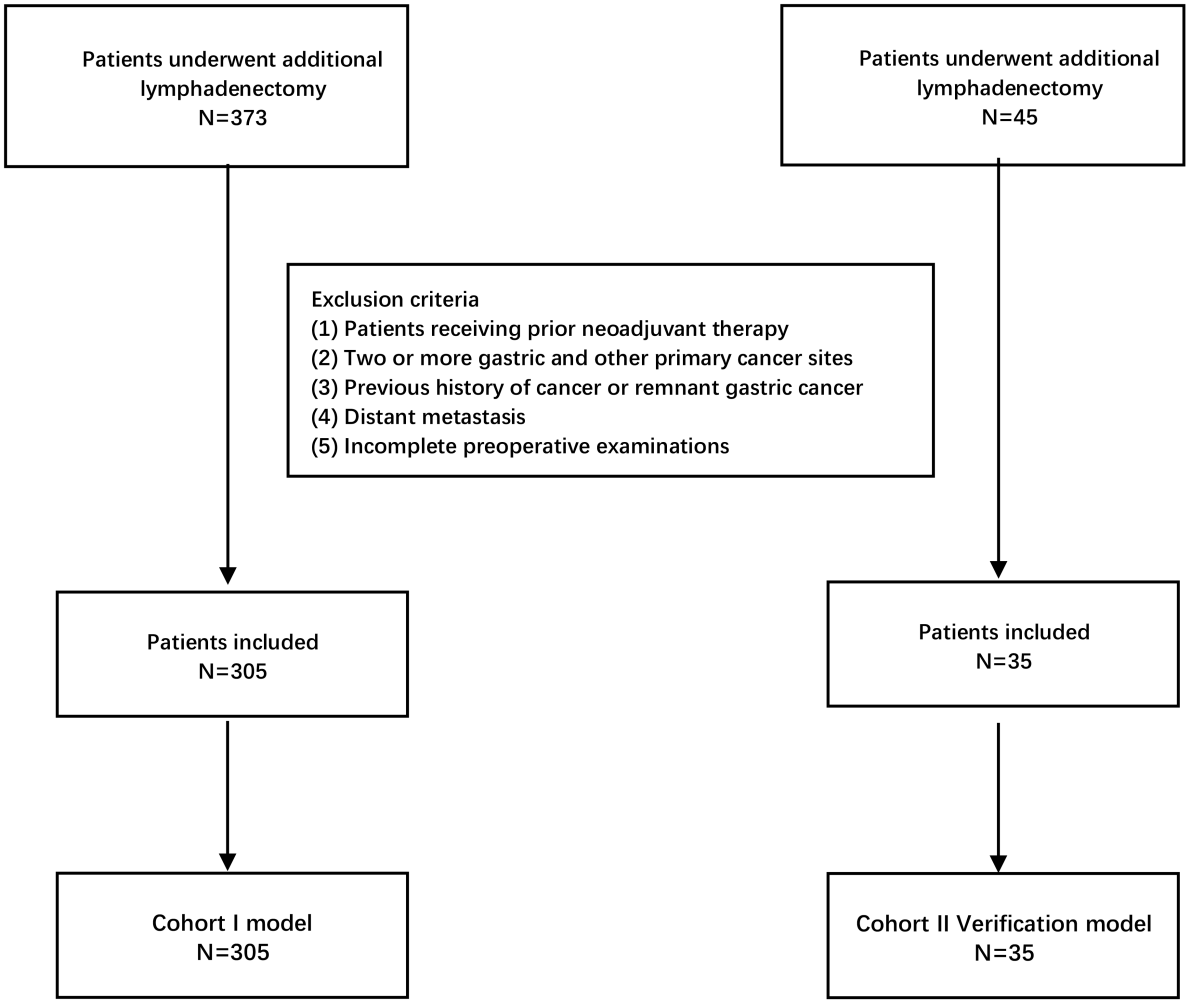
Flowchart of patients included in the study for construction models and external validation models according to the inclusion and exclusion criteria.

The authors retrieved EGC patients who only received additional gastrectomy from the electronic medical record system of the Sixth Affiliated Hospital of Sun Yat-Sen University (Guangzhou, China). All patients were recruited from January 2012 to March 2021. After screening, a total of 373 records were found, and 68 patients met any of the exclusion criteria; then, 305 cases with pathologically confirmation of T1a/T1b stage were included in the study and underwent additional gastrectomy with systemic lymphadenectomy (D2) (**Figure 1**). The exclusion criteria in this study were as follows: (1) patients who have received previous neoadjuvant therapy, (2) patients that present two or more gastric and/or other primary cancer type, (3) patients’ previous history of cancer or remnant gastric cancer, (4) patients with distant metastasis, and (5) incomplete preoperative examinations (variables with >25% of missing information), including blood analysis, gastroscopy pathological reports, and/or pathological results. These exclusion criteria were used for both cohort I and cohort II by the ML models. For the external validation, a cohort of 35 patients who underwent additional gastrectomy with standard lymphadenectomy at the University Hospital Virgen del Rocio (Seville, Spain) between January 2014 and December 2020 was recruited (**Figure 1**).

### Clinicopathological evaluation

The medical records for blood analysis, gastroscopy, and pathological reports for each patient were reviewed for the analysis. From the blood analyses data were gathered tumor markers such as CEA, CA199, CA125, CA153, and AFP. Gastroscopy data were collected from the report, which included the location of the tumor. The pathological results provided information about invasion depth (T1a/T1b), histologic type, Lauren classification, tumor size, and ulceration. The clinical characteristics of the patients, including sex, age, body mass index (BMI), and personal pathological history were also collected.

### Statistical analysis and ML models

#### Association analysis

According to the clinicopathological results, the univariate analysis was performed on all variables; all data sets were divided into two groups according to the lymph nodes positiveness. Association analysis was applied to all variables individually, categorical variables with expected frequency greater than 5 in the LNM group and the non-LNM group were tested by chi-square test, and categorical variables with expected frequency less than 5 in the LNM group or non-LNM group were tested by Fisher’s exact test. Continuous variables were tested by the T student test (the *p*-value greater than 0.05 in Shapiro–Wilk test and Levine’s test) and the Mann–Whitney test. The chi-square test or Fisher’s exact test was also used for tumor markers after categorization into binary variables using the following cutoff points set as normal range (37 U/ml for CA19-9, 5 ng/ml for CEA, 35 U/ml for CA125, 32.4 U/ml for CA153, and 8.78 ng/ml for AFP) (32).

#### ML models

After a comprehensive review of different ML prediction algorithms reported in the literature, compared the scalable, flexible, accurate, and relatively fast, five types of supervised ML classifiers were selected to provide for the establishment the prediction model in EGC (33–37). These models were the logistic regression classifier (LRC), linear support vector classifier (Linear SVC), Gaussian process classification (GPC), and two gradient boosting methods extreme gradient boosting (XGBoost) and light gradient boosting machine (LightGBM).

LRC is a classification model rather than regression model, which is a simple and more efficient method for binary and linear classification problems; it is a classification model that is very easy to realize and achieves excellent performance with linearly separable classes (38). Linear SVC was performed to obtain method based on support vector classifier (SVM). SVM is a widely used alternative to softmax for classification and is used for both linear and nonlinear classification by changing the kernel functions utilized (39). GPC can naturally give predicted probabilities for classification problems that require tuning of the kernel functions (40). It was used for complex non-parametric ML algorithms for classification and regression (41). XGBoost and LightGBM were considered among the most recent and efficient ML-based prediction algorithms (42). The XGBoost model, which can handle both regression and classification problems, is widely used by data scientists to achieve state-of-the-art results (43). LightGBM is a gradient learning framework based on the decision tree and the idea of boosting (44). Its major difference from the XGBoost model is that it uses histogram-based algorithms to speed up the training process, reduce memory consumption, and employ a leaf-wise growth strategy with depth constraints (37). The original codes of these five algorithms, which were performed in this study, were based on Python 3.9 and scikit-learn 1.0 (45).

#### Feature selection and construction the ML methods

For ML approach, all features included in the model were determined by the Akaike Information Criterion (AIC), Bayesian Information Criterion (BIC), and the Least Absolute Shrinkage and Selection Operator (LASSO), which were widely used for finding the best features for models (46, 47). According to the previous study (48), all variables were included for feature selection in the LASSO binary logistic regression model, in the AIC scores, and in the BIC scores for all possible combinations, which with *p* < 0.15 in the univariable analysis were predefined as the cutoff and the factors were reported from previous study in the LASSO binary logistic regression model, in the AIC scores, and in the BIC scores for all possible combinations. The final features were applied to establish ML models depending on these three methods (AIC, BIC, and LASSO). The statistical analyses were performed using SPSS^®^ version 26 (IBM SPSS Statistics for Macintosh) and R Studio (Integrated Development for R. RStudio, PBC, Boston, MA, version 4.0.5).

All selected categorical features were transformed into dummy variables. Then, all features were used to construct the ML models to predict LNM. All models used fivefold cross-validation on both cohort I and cohort II. All models were evaluated by the receiver operating characteristic curve (AUC) and optimized by the grid search; the Bayesian method was used to improve the ability of model. For LRC and Linear SVC models, the importance of features was calculated by their weight coefficients. For XGBoost and LightGBM, the importance of features was also plotted. All models were constructed and analyzed by Python (version 3.9.4). All files used for model construction have been placed in the supplement.

### External validation

All ML models were verified by external validation data and accuracy; AUC, Brier score, F1 score sensibility, specificity, and 95% ICs were estimated using the bootstrap method. Other bioinformatic approaches such as confusion matrices, ROC curves, and calibration curves were used in the present analysis. The groups that exhibited a high-risk were established by predictive probability, and their relative odds ratios were calculated.

## Results

### Clinicopathological variables associate with lymph node metastasis

The primary cohort (cohort I) included a total of 305 patients, of whom 69 patients (22.6%) had LNM according to the 8th edition of the American Joint Committee on Cancer (AJCC) staging system (49). The classification of tumor size was based on the eCura system of the Japanese Gastric Cancer Treatment Guidelines ed. 2018 (50). The tumor size was divided into three groups (≤ 2cm, 2–3 included, > 3cm). Their demographic and clinicopathological characteristics are shown in **Table 1**. In univariable analysis (in the association analysis), “age” was the only continuous variable that showed statistically significant differences between both groups (*t* = 2.64, *P* = 0.009). After categorization, this variable was divided into five groups based on the risk of cancer associated to age from National Cancer Institute of US (< 30 years, 30–40 years, 40–50 years, 50–60 years, and > 60 years) (51). The chi-square test showed statistically significant differences between all five groups (χ^2^ = 20.991, *P* < 0.001). The biomarkers such as CEA (*U* = 9006, *P* = 0.178) and CA125 (*U* = 7123.5, *P* = 0.114) met the variable filter criteria, but their binary form (normal *vs.* high) were not statistically significant (*P* = 1.0 and *P* = 0.428, respectively). Other categorized variables such as invasion depth (χ^2^ = 17.377, *P* < 0.001), histologic type (χ^2^ = 7.715, *P* = 0.005) and LAUREN classification (χ^2^ = 11.260, *P* = 0.005) were statistically significant, and the presence of ulcer presented a high trend toward significance (χ^2^ = 3.741, *P* = 0.053). Nevertheless, tumor size (χ^2^ = 3.590, *P* = 0.166) and tumor location (χ^2^ = 4.260, *P* = 0.119) exhibited no association with LNM.

**Table 1:** Clinicopathologic characteristics of patient samples included in the present study.

Variable	LNM negative (*N* = 236)	LNM positive (*N* = 69)	*P*-value
**Age** (year, mean±std)	59.0 ± 11.4	54.87 ± 12.5	**0.009****^a^**
**Gender**			0.697^C^
Male (*n*, %)	135 (57.21%)	37 (53.62%)	
Female (*n*, %)	101 (42.79%)	32 (46.38%)	
**BMI** (mean ± std)	22.69 ± 3.44	22.62 ± 2.83	0.881^a^
**DM**			0.764^d^
Yes (*n*, %)	12 (5.08%)	4 (5.79%)	
No (*n*, %)	224 (94.92%)	65 (94.21%)	
**HTA**			0.321^C^
Yes (*n*, %)	31 (13.14%)	13 (18.84%)	
No (*n*, %)	205 (86.86%)	56 (81.16%)	
**Tumor location**			0.119^C^
Fundus (*n*, %)	31 (13.14%)	3 (4.35%)	
Body (*n*, %)	52 (22.03%)	18 (26.09%)	
Antrum (*n*, %)	153 (64.83%)	48 (69.56%)	
**Depth of invasion**			**<0.001^C^**
T1a (*n*, %)	131 (55.51%)	18 (26.09%)	
T1b (*n*, %)	105 (44.49%)	51 (73.91%)	
**Histologic type**			**0.005^C^**
Undifferentiated type (*n*, %)	147 (62.29%)	56 (81.16%)	
Differentiated type (*n*, %)	89 (37.71%)	13 (18.84%)	
**LAUREN classification**			**0.004^C^**
Diffuse type (*n*, %)	93 (39.41%)	34 (49.28%)	
Intestinal type (*n*, %)	95 (40.25%)	13 (18.84%)	
Mixed type (*n*, %)	48 (20.34%)	22 (31.88%)	
**Tumor size**			0.166^C^
2–3(included) cm (*n*, %)	58 (24.58%)	19 (27.54%)	
>3 cm (*n*, %)	38 (16.10%)	17 (24.64%)	
≤2cm (*n*, %)	140 (59.32%)	33 (47.82%)	
**Ulceration**			0.053^C^
Negative (*n*, %)	165 (69.92%)	39 (56.52%)	
Positive (*n*, %)	71 (30.08%)	30 (43.48%)	
**AFP** (median, range)	2.47 (0.95-14.37)	2.74 (0.84-107.97)	0.279^b^
**CA125** (median, range)	9.55 (2.7-130.7)	10.6 (3.1-191.7)	0.114^b^
**CA153** (median, range)	7.2 (1.9-27.1)	10.6 (3.1-19.6)	0.858^b^
**CA199** (median, range)	4.90 (2-115.14)	5.11 (2.0-338.54)	0.743^b^
**CEA** (median, range)	2.08 (0.51-23.59)	1.87 (0.53-9.88)	0.178^b^

a Independent two-sample t-test.

b Mann–Whitney U test.

c *X* ^2^ test with Yates’ continuity correction.

d Fisher’ s exact test.

The bold values means these variables show statistically significant differences between both groups.

### Selected variables

**Figure 2:**
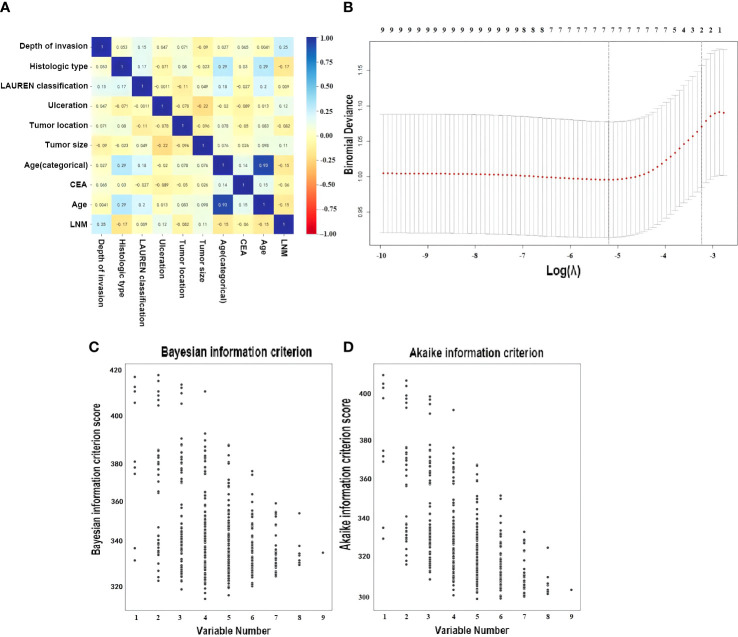
Optimal variable combination selection. **(A)** Correlation matrix of variables. **(B)** Result by Least Absolute Shrinkage and Selection Operator (LASSO). Here, the partial likelihood deviance (binomial deviance) curve was plotted in log(λ) scale. Dotted vertical lines were drawn at the values of log(λ) with minimum mean squared error (MSE) and the maximum log(λ) of one SE of the minimum MSE. The best features were selected with minimum mean squared error (MSE) from the five cross-validation folds, with lambda value 0.00558, log(λ) is −5.19. One SE of the minimum MSE with lambda value 0.03936, log(λ) is −3.24. **(C)** Dot plot performed by Bayesian Information Criterion (BIC) for all possible models (disregarding potential transformations and interactions) employing none, any or all of the seven selected risk factors, a lower BIC indicates a better fit (52). **(D)** Dot plot performed by Akaike Information Criterion (AIC), a lower AIC indicates a better fit (53).

A total of seven variables were included as potential risk factors in the prediction model, which the *p*-values in univariable analysis were less than 0.15 (**Table 1** and **Figure 2A**). CEA and tumor size have been reported from the previous study, which were related with LNM in EGC (54, 55), but CA125 was discarded from the model by lack of data in the cohort II. In the LASSO method, the including variables were exhibited a minimum mean squared error (MSE) by five cross-validation folds, which were the invasion depth, histologic type, ulceration, tumor location, tumor size, Lauren classification, and age. The variables included which with standard error of MSE contained the age and invasion depth (**Figure 2B**). There are five variables in the group; the minimum AIC score was 299.08 with five variables, which were the depth of invasion, histologic type, the presence of ulcer, tumor size, and age (**Figure 2C**) and, in the minimum, BIC score was 301.07 and was obtained with four variables, which were the depth of invasion, the presence of ulcer, tumor size, and age (**Figure 2D**). Finally, the features selected with minimum mean squared error (MSE) in LASSO were applied to establish the prediction ML models. Finally, seven variables, namely, age, tumor location, histologic type, the LAUREN classification, tumor size, invited depth, and ulceration (positive/negative), were included in at least one of these methods. These seven variables were used to training the ML models.

Once the variables were selected with LASSO, **Table 2** was assessed to compare detailed clinic-pathological characteristics between the cohort I and cohort II groups. Both cohort I and cohort II had a ratio of LNM negative/positive similar, 3.42 and 3.38, respectively.

**Table 2:** Clinicopathologic characteristics for established the prediction model between cohort I and cohort II.

Variable	Cohort I		Cohort II	
	LNM negative (*N* = 236)	LNM positive (*N* = 69)	LNM negative (*N* = 27)	LNM positive (*N* = 8)
**AGE** (years)				
age < 30 (*n*%)	1 (0.42)	5 (7.25)	0 (0.00)	0 (0.00)
age 30–40 (*n*%)	20 (8.47)	3 (4.35)	1 (3.70)	0 (0.00)
age 40–50 (*n*%)	28 (11.86)	16 (23.19)	2 (7.41)	1 (12.50)
age 50–60 (*n*%)	75 (31.78)	22 (31.88)	1 (3.70)	1 (12.50)
age > 60 (*n*%)	112 (47.47)	23 (33.33)	23 (85.19)	6 (75.00)
**TUMOR LOCATION**				
Fundus (*n*%)	31 (13.14)	3 (4.35)	0 (0.00)	2 (25.00)
Body (*n*%)	52 (22.03)	18 (26.09)	12 (44.44)	2 (25.00)
Antrum (*n*%)	153 (64.83)	48 (69.56)	15 (55.56)	4 (50.00)
**HISTOLOGIC TYPE**				
Undifferentiated (*n*%)	147 (62.29)	56 (81.16)	12 (44.44)	4 (50.00)
Differentiated (*n*%)	89 (37.71)	13 (18.84)	15 (55.56)	4 (50.00)
**LAUREN**				
Diffuse (*n*%)	93 (39.41)	34 (49.28)	7 (25.93)	3 (37.50)
Intestinal (*n*%)	95 (40.25)	13 (18.84)	20 (74.07)	3 (37.50)
Mixed (*n*%)	48 (20.34)	22 (31.88)	0 (0.00)	2 (25.00)
**TUMOR SIZE (cm)**				
2–3 (include) (*n*%)	58 (24.58)	19 (27.54)	7 (25.93)	5 (62.50)
> 3 (*n*%)	38 (16.10)	17 (24.64)	10 (37.04)	3 (37.50)
≤ 2 (*n*%)	140 (59.32)	33 (47.82)	10 (37.04)	0 (0.00)
**DEPTH OF INVASION**				
T1a (*n*%)	131 (55.51)	18 (26.09)	8 (29.63)	0 (0.00)
T1b (*n*%)	105 (44.49)	51 (73.91)	19 (70.37)	8 (1.00)
**ULCERATION**				
Negative (*n* %)	165 (69.92)	39 (56.52)	11 (40.74)	1 (12.50)
Positive (*n* %)	71 (30.08)	30 (43.48)	16 (59.26)	7 (87.50)

### ML models can predict lymph node metastasis

**Figure 3:**
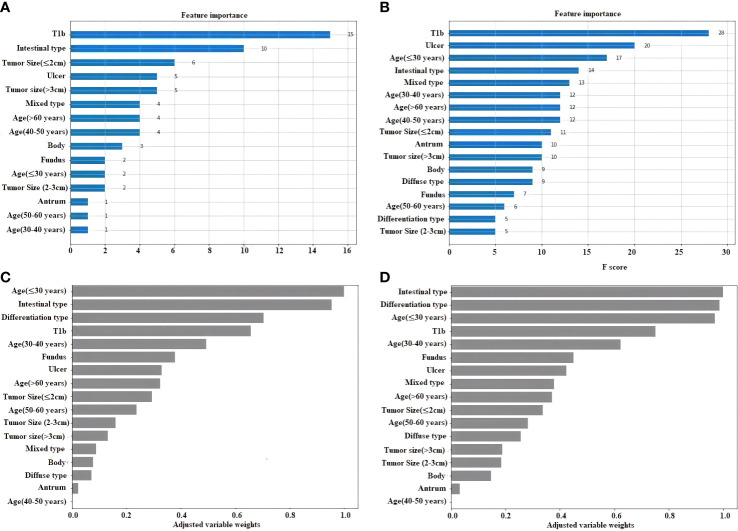
Feature importance plot for the 4 ML. **(A)** Light gradient boosting machine classifier (LightGBM). **(B)** Extreme gradient boosting classifier (XGBoost). **(C)** Logistic regression classifier. **(D)** Linear support vector machine classifier (Linear SVC).

The statistical weigh of the different variables for the light gradient boosting machine classifier (LightGBM), extreme gradient boosting classifier (XGBoost), LRC, and linear support vector machine classifier (Linear SVC) are shown in **Figure 3**. Tumors invaded the submucosal (T1b), intestinal type, age < 30, and the presence of ulcer were the four factors with the highest statistical power to establish these four models.

**Figure 4:**
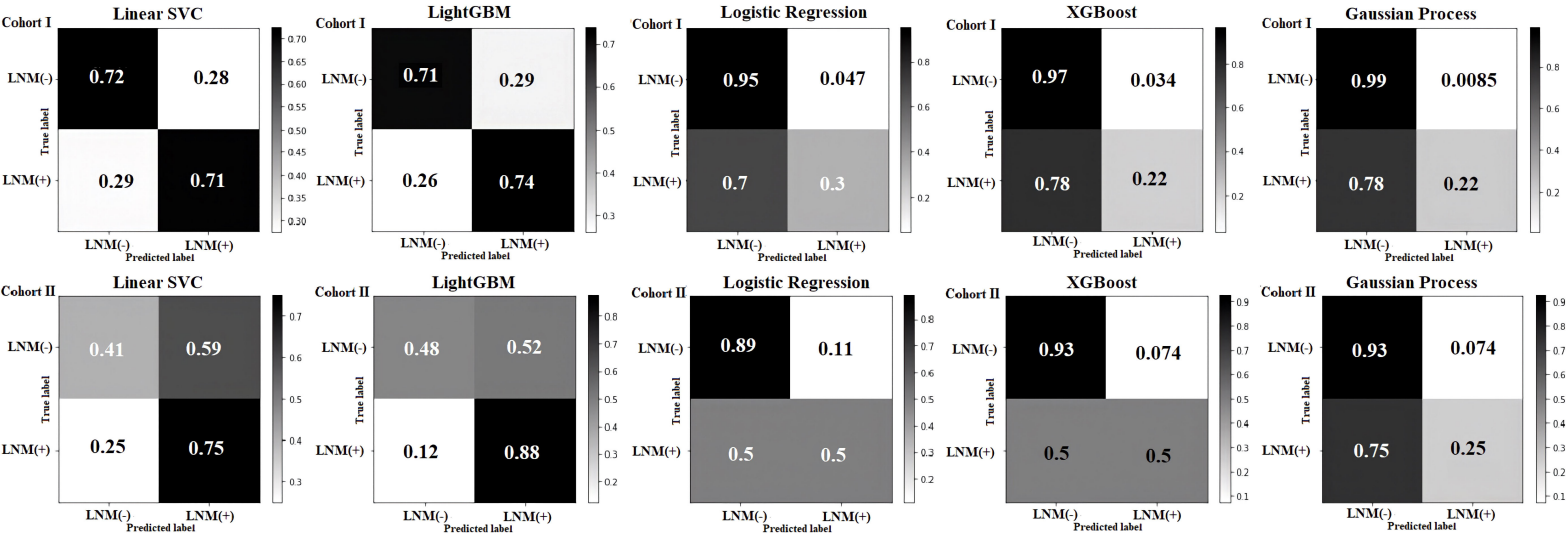
Confusion matrix of the cohort I and the cohort II in five machine learning models. In each subplot, the specificity, false positive rate (FPR), false negative rate (FNR), and sensibility were shown from top left to bottom right, respectively.

The confusion matrices for the five classifiers in the cohort I and cohort II with the percentage of their true label are displayed in **Figure 4**. This corresponds to specificity, false positive rate (FPR), false negative rate (FNR), and sensibility in each subplot. Both Linear SVC and LightGBM presented a better sensibility in the models; the Linear SVC showed a robust performance in sensibility, 0.71 in cohort I and 0.75 in cohort II. The logistic regression, XGBoost, and the Gaussian process classifier performed a better specificity. Concerning the sensibility, in the Logistic Regression and XGBoost were improved in cohort II, both with a sensibility of 0.5, equal a completely random decision. The Gaussian process classifier was the most stable model in these five models, and the best performance in specificity, with 0.99 in the cohort I, and 0.93 in the cohort II.

**Figure 5:**
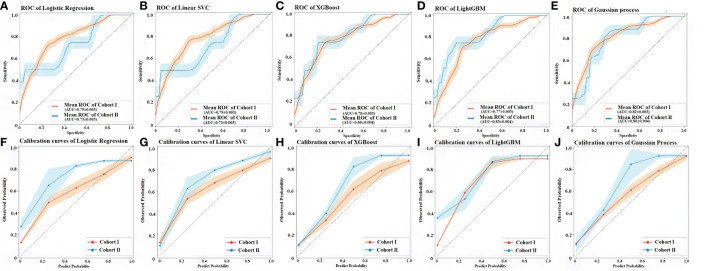
Discrimination and calibration performance of the 5 models. **(A)** ROC curves of the Logistic regression classifier in the cohort I and cohort II, respectively (AUC=0.788, 95% CI 0.785–0.790 *versus* 0.732, 95% CI 0.727–0.738). **(B)** ROC curves of the linear support vector machine classifier (Linear SVC) in the cohort I and cohort II, respectively (AUC=0.786, 95% CI 0.783–0.789 *versus* 0.736, 95% CI 0.731–0.741). **(C)** ROC curves of the in the extreme gradient boosting classifier (XGBoost) in the cohort I and cohort II, respectively (AUC = 0.781, 95% CI 0.778–0.784 *versus* 0.804, 95% CI 0.799–0.809). **(D)** ROC curves of the Light gradient boosting machine classifier (LightGBM) in the cohort I and cohort II, respectively (AUC = 0.766, 95% CI 0.763–0.769 *versus* 0.830, 95% CI 0.826–0.835). **(E)** ROC curves of the Gaussian process classification in the cohort I and cohort II, respectively (AUC = 0.816, 95% CI 0.813–0.819 *versus* 0.803, 95% CI 0.799–0.808). The light orange area and blue area represent the 95% CIs in cohort I and cohort II, respectively. 500 Bootstrap resamples were used to calculate a relatively corrected AUC and 95% CI. Calibration curves of five models in the cohort I and cohort II are shown in figures from **(F–J)** The 45° dashed line represents a perfect prediction, the orange lines represent the predictive performance of the model in the cohort I, and the blue lines represent the predictive performance of the model in the cohort II. The closer the dotted line to the ideal line, the better the predictive accuracy of the model is (56). AUC, area under the curve; CI, confidence interval; ROC, receiver operating characteristic.

The discrimination and calibration of the five models in the cohort I and cohort II were shown in **Figure 5**. For testing models of ML, each model had better ability to the prediction, the area under the curve (AUC) values of all algorithms were closed to 0.8 between the cohort I and cohort II, even the Gaussian process classification had exceeded this value in both set (0.816, 95% CI 0.813–0.819 *vs.* 0.803, 95% CI 0.799–0.808). However, compared with the different values of AUC between the cohort I and cohort II for all models, the XGBoost (0.781 *vs.* 0.804) and the Gaussian process classification (0.816 *vs.* 0.803) had tiny difference in both sides. It meant that these two models had the almost same ability for the prediction in cohort I and cohort II (**Figures 5A, E**). The 95% CI of the calibration belt in both cohort I and cohort II did not cross the diagonal bisector line, which suggests that the prediction models had a strong concordance between both groups and further indicates the five models demonstrate an accurate prediction potential in both groups. The XGBoost and the Gaussian process classification were closer the dotted line to the ideal line, these two models had the better the predictive accuracy (**Figures 5F–J**).

**Table 3** shows the prediction performance of five ML classifiers for cohort I and cohort II. The XGBoost classifier and Gaussian process classification demonstrated the best performance due to there was a little difference between the cohort I and cohort II: the cohort I´s specificity 96.7% (95% CI 96.5–96.8%) and 99.1% (95% CI 99.1–99.2%); accuracy 79.6% (95% CI 79.4–79.8%) and 81.5% (95% CI 81.3–81.7%); AUC 78.1% (95% CI 77.8–78.4%) and 81.6% (95% CI 81.3–81.9%), the cohort II´s specificity 92.6% (95% CI 92.3–92.8%) and 92.6% (95% CI 92.3–92.8%); accuracy 82.6% (95% CI 82.3–82.8%) and 77.1% (95% CI 76.8–77.4%); AUC 80.4% (95% CI 79.9–80.9%) and 80.3% (95% CI 79.9–80.8%), respectively. The sensibility and F1 score values were also demonstrated in this table. The F1 score can be interpreted as a harmonic mean of the precision and recall, where an F1 score reaches its best value at 1 and worst score at 0 (57) although, in these five models, the F1 score was already between in 0.33 and 0.57. A brier score was a way to verify the accuracy of a probability forecast. A probability forecast refers to a specific event. The best possible Brier score is 0, for total accuracy. The lowest possible score is 1, which means the forecast was wholly inaccurate (58). In this study, all of the models had the Brier score, which was less than 0.25.

**Table 3:** Validation performance for the prediction of LNM of EGC by using five machine learning classifiers.

Machine learning	Sensibility (95% CI)	Specificity (95% CI)	F1 Score (95% CI)	Accuracy (95% CI)	AUC (95% CI)	Brier (95% CI)
**Linear SVC**						
Cohort I	0.711 (0.707–0.716)	0.727 (0.724–0.729)	0.538 (0.534–0.542)	0.723 (0.720–0.725)	0.786 (0.783–0.789)	0.207 (0.204–0.211)
Cohort II	0.748 (0.740–0.755)	0.408 (0.404–0.412)	0.398 (0.393–0.403)	0.486 (0.482–0.489)	0.736 (0.731–0.741)	0.225 (0.223–0.227)
**Logistic Regression**						
Cohort I	0.302 (0.297–0.308)	0.955 (0.953–0.956)	0.413 (0.407–0.419)	0.806 (0.804–0.808)	0.788 (0.785–0.790)	0.189 (0.186–0.191)
Cohort II	0.500 (0.492–0.509)	0.890 (0.887–0.892)	0.531 (0.524–0.538)	0.798 (0.795–0.801)	0.732 (0.727–0.738)	0.235 (0.232–0.237)
**XGBoost**						
Cohort I	0.215 (0.210–0.220)	0.967 (0.965–0.968)	0.323 (0.317–0.329)	0.796 (0.794–0.798)	0.781 (0.778–0.784)	0.145 (0.143–0.146)
Cohort II	0.500 (0.492–0.509)	0.926 (0.923–0.928)	0.568 (0.561–0.575)	0.826 (0.823–0.828)	0.804 (0.799–0.809)	0.172 (0.171–0.174)
**LightGBM**						
Cohort I	0.739 (0.734–0.743)	0.708 (0.705–0.711)	0.540 (0.536–0.544)	0.714 (0.712–0.717)	0.766 (0.763–0.769)	0.234 (0.233–0.236)
Cohort II	0.880 (0.874–0.886)	0.478 (0.474–0.482)	0.480 (0.475–0.485)	0.566 (0.563–0.569)	0.830 (0.826–0.835)	0.245 (0.243–0.247)
**Gaussian Process**						
Cohort I	0.214 (0.209–0.219)	0.991 (0.991–0.992)	0.344 (0.337–0.350)	0.815 (0.813–0.817)	0.816 (0.813–0.819)	0.139 (0.138–0.140)
Cohort II	0.254 (0.246–0.262)	0.926 (0.923–0.928)	0.333 (0.324–0.342)	0.771 (0.768–0.774)	0.803 (0.799–0.808)	0.185 (0.184–0.187)

**Figure 6:**
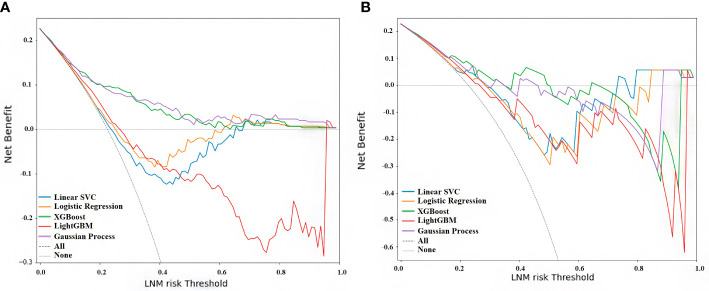
Decision curve analysis for all five models. **(A)** Curve of cohort (I) **(B)** Curve of cohort II. The x-axis measures the net benefit, and the y-axis shows the LNM risk threshold. The blue line represents the linear support vector machine classifier (Linear SVC), the orange line the logistic regression classifier, the green line the extreme gradient boosting classifier (XGBoost), the red line the light gradient boosting machine classifier (LightGBM), the purple line the Gaussian process classification, the gray solid line the assumption that no patient with LNM, and the dashed line represents all patients with LNM (59).

The decision curve of the XGBoost and Gaussian Process Classification models had a more comprehensive net benefit threshold probability range in the cohort I, although these were no statistical differences in the cohort II (**Figure 6**). Analysis showed that when the predictive criticism was > 0 in the XGBoost model and Gaussian process classification in the cohort I, the models added more net benefit than “no patient with LNM” or “all patients with LNM” scheme (**Figure 6A**). The predictive criticism ranged from 0 to 0.357 of the XGBoost model and 0 to 0.293 of the Gaussian process classification in the cohort II, the models added more net benefit than “no patient with LNM” or “all patients with LNM” scheme (**Figure 6B**).

Subsequently, the predicted probability was categorized as low, medium, and high risk. **Table 4** shows the odds ratio (OR) value of LNM prediction for each model. When comparing the different levels of risk, Linear SCV classifier, XGBoost classifier, and Gaussian process classification showed the highest capacity for the prediction due to the positive gradient increasing in different levels. The medium risk of Linear SCV classifier was 3.5 times higher than the low risk, and the high risk was seven times than the low risk. The Gaussian Process has 5.46 and 16.67 times comparing the medium and high risk with low risk. Even though the medium risk of XGBoost showed no statistically significant increasing compared with the low risk (1.67 *vs.* 1). LRC demonstrated the negative gradient comparing the high and medium risk (4.5 *vs.* 4.8), and LightGBM showed the negative gradient in medium and low risk (0.89 vs 1).

**Table 4:** Odds ratio and confidence intervals between different risk group in five machine learning classifiers.

OR values (95% CI)	Linear SVC	Logistic regression	XG Boost	Light GBM	Gaussian process
**Low risk**	1 (reference)	1 (reference)	1 (reference)	1 (reference)	1 (reference)
**Medium risk**	3.5 (3.15–3.89)	4.8 (4.32–5.34)	1.67 (1.54–1.81)	0.89 (0.79–0.99)	5.46 (4.94–6.03)
**High risk**	7.0 (6.35–7.71)	4.5 (4.08–4.96)	10.0 (9.26–10.79)	4.0 (3.63–4.41)	16.67 (15.10–18.39)

Range of predicted probability: low risk (0–0.25); medium risk (0.25–0.5); high risk (>0.5).

## Discussion

With the development of minimally invasive endoscopic technology, ESD is the gold standard to treat the EGC (7, 50, 60), due to the benefit such as minor trauma, quick recovery, and a better quality of life could be improved after the treatment (61, 62). However, the LNM is a problem that depends on whether receive or not an additional lymphadenectomy. The traditional methods of predicting LNM could have certain limitations, in recent studies the EGC patients with only the evaluation of clinicopathological characteristics after ESD needed to perform additional surgery due to having a high risk of LMN; however, actually, the risk of LNM was approximately 10% after the lymphadenectomy (19, 20, 63). Therefore, a good predictive method can predict LNM in nearby 80% and help to reduce unnecessary surgery and improve the patient’s quality of life. ML had been used broadly in medicine, since it can help to improve the accuracy of clinical prediction (28, 64, 65). In this study, we found that the ML models were the most important benefit of improving predictive accuracy to detect the LNM in EGC.

According to the feature selection, we found that the risk factors related to LNM such as age; the presence of ulceration, tumor size, and depth of invasion; the histologic tumor type; the tumor location; and Lauren classification were common in each model (AIC, BIC, and LASSO) (**Figure 2**). This is almost consistent with the ranking of variables importance in the results of ML models, although the order was different (**Figure 3**). Previous studies had been considered that these factors were related to the LNM in EGC (13, 66). On the other hand, the age was the risk that was included in these prediction models (**Figure 3**), although the age was not contained in the traditional evolution scale (50), but age-related studies involving many carcinoma patients have yielded some relevant results (67, 68). Perhaps, in the future, based on the ML models, we can find more factor combinations that would be constructed the optimized group that influences the LNM in EGC. This fact can provide a new solution to find the related factors and design new ML models in clinical research for prediction.

Another point in this study was the use of ML for the prediction of LNM. Here, we found that the Linear SVC and Light gradient boosting classifier (LightGBM) were the best models to detect the actual positive cases, although the rest three models presented excellent abilities to detect the actual negative cases (**Figure 4**). According to the predicted probability, the XGBoost classifier and Gaussian process classification had the best predictive accuracy of the model than the others. This is probably due to the random sampling results that were closer to the ideal line (**Figure 5**). Furthermore, they had a more comprehensive net benefit threshold probability range in the cohort I, which that meant for the patient with LNM who was predicted by XGBoost model and Gaussian process; the additional treatment could be had more benefit for them (**Figure 6**). In the predicted probability among different risk groups, the Linear SVC, XGBoost classifier, and Gaussian process had a certain degree of discrimination. The OR value was obviously increased among low, medium, and high risk, which were applied with the Linear SVC, and Gaussian process. This means that these two models are better to detect the risk in different groups (**Table 4**). Thus, as can be observed, each model has its own characteristics and advantages in prediction, but Gaussian Process shows the best comprehensive predictive ability in this study. Perhaps, for the prediction of the LNM in EGC, we could combine multiple models to increase prediction ability. Xiao Y. et al. demonstrated that the ML methods have been more and more widely used in cancer prediction. However, no individual method exceeded the others, and a combination of models could imply an optimal final prediction (69).

It is undeniable that this study also has certain limitations. First, the model was constructed using a retrospective cohort; therefore, a prospective data set could be appropriate to improve the ability of the prediction model; perhaps we can find more risks that could be related to LNM. In addition, all preoperative examination results were obtained from reports; therefore, information bias was unavoidable. This study has been performed with a limited sample size, especially cohort II. However, results differed slightly between the cohort I and cohort II, which implies not only a different origin (China and Spain) but also a different ethnicity. In future work, we will make a prospective trial that includes more variables, such as biomarkers, and supplement with more predictive models to improve the prediction ability.

In conclusion, we established five commonly used ML models to predict LNM in EGC; according to our results, machine learning can be used to detect high-risk LNM in EGC, especially the Gaussian Process Classification had the best comprehensive predictive ability. This could be applied to indicate that additional lymphadenectomy is necessary after the endoscopic resection in EGC. From another point of view, machine learning could provide a new solution to find the related factors in clinical research for prediction of LNM in EGC.

## Data availability statement

The original contributions presented in the study are included in the article/**Supplementary Material**. Further inquiries can be directed to the corresponding authors.

## Author contributions

Study concept and design: TY, JM-U, JL, and SM-C; Acquisition of data: TY, IA, and XJ; Build Models’ code: CL; Analysis and interpretation: TY, WL, YX, XJ, and YZ; Study supervision: SM-C and ZY. All authors contributed to the article and approved the submitted version.

## Funding

This work was supported by grants from the International Postdoctoral Exchange Fellowship Program 2020 by Human Resources and Social Security Department of Guang Dong Province support; Guangdong Provincial Department of Science and Technology 2021 Guangdong International, Hong Kong, Macao and Taiwan High-end Talent Exchange Overseas Famous Teacher Project (version number: 109123037043, project name: ICG Mapping in lymphadenectomy of gastric cancer); the National Key Clinical Discipline, the National Natural Science Foundation of China (Grant Nos 81772594, Z.Y.; 81802322, H.C. and 81902949, J.H.), the Science and Technology Program of Guangzhou (Grant No. 201803010095, Z.Y.), and the Natural Science Foundation of Guangdong Province, China (Grant Nos 2020A1515011362, Z.Y. and 2022A1515010262, Z.Y.).

## Acknowledgments

This study was supported by Department of Gastrointestinal Surgery, The Sixth Affiliated Hospital of Sun Yat-sen University, Guangzhou, China, and the Unit of Innovation in Minimally Invasive Surgery, Department of General and Digestive Surgery, University Hospital “Virgen del Rocio”, Sevilla, Spain.

## Conflict of interest

The authors declare that the research was conducted in the absence of any commercial or financial relationships that could be construed as a potential conflict of interest.

## Publisher’s note

All claims expressed in this article are solely those of the authors and do not necessarily represent those of their affiliated organizations, or those of the publisher, the editors and the reviewers. Any product that may be evaluated in this article, or claim that may be made by its manufacturer, is not guaranteed or endorsed by the publisher.
